# Silicon Mode-Selective Switch via Horizontal Metal-Oxide-Semiconductor Capacitor Incorporated With ENZ-ITO

**DOI:** 10.1038/s41598-019-54332-6

**Published:** 2019-11-28

**Authors:** Weifeng Jiang, Jinye Miao, Tao Li

**Affiliations:** College of Electronic and Optical Engineering, Nanjing University of Posts and Telecommunications, Nanjing, 210023 China

**Keywords:** Fibre optics and optical communications, Integrated optics

## Abstract

A silicon mode-selective switch (MSS) is proposed by using a horizontal metal-oxide-semiconductor (MOS) capacitor incorporated with the epsilon-near-zero (ENZ) indium-tin-oxide (ITO). The carrier concentration of the double accumulation-layers in ITO can be adjusted via the applied gate-voltage to achieve the desired switching state. The MOS-type mode of the central MOS-capacitor based triple-waveguide coupler is introduced and optimised by using the full-vectorial finite element method to switch the “OFF” and “ON” states. The thickness of the accumulation layer and the optimal design are studied by using the 3D full-vectorial eigenmode expansion method. The optimised quasi-TE_0_ and quasi-TE_1_ modes based MSSes are with the extinction ratios of 28.52 dB (19.05 dB), 37.29 dB (17.8 dB), and 37.29 dB (23.7 dB), at “OFF” (“ON”) states for the accumulation-layer thicknesses of 1.5, 5.0, and 10.0 nm, respectively. The operation speed can achieve to be 6.3 GHz, 6.2 GHz, and 6.2 GHz for these three accumulation-layer thicknesses, respectively. The performance of the proposed MSS with a 2.5 V gate-voltage is also studied for preventing the oxide breakdown. The proposed MSS can be applied in the mode-division-multiplexing networks for signal switching and exchanging.

## Introduction

Mode division multiplexing (MDM) technology is of great promise to overcome the communication bottleneck and to achieve a dramatic capacity-enhancement for optical transmission networks^1,2^. Silicon photonics show attractive characteristics to realise compact, low-cost, and CMOS-compatible optical devices^3^. To build on-chip MDM systems, various silicon based building blocks have been demonstrated, including the mode (de)multiplexers [(De)MUXs]^4,5^, multimode power-splitters^6^, mode filters^7^, multimode bent waveguides/crossings^8,9^, and mode-selective switches (MSSes)^10–12^. Among these devices, an MSS is the basic and critical component for flexible mode-routing and switching to achieve a reconfigurable MDM network.

Recently, a few approaches have been reported to build a silicon MSS for reconfigurable MDM networks, including the micro-ring resonators (MRRs)^13^, Mach–Zehnder interferometers (MZIs)^14–16^, multimode interference (MMI) couplers^17,18^, and triple-waveguide couplers (TWCs)^19–21^. Stern *et al*. firstly demonstrated a 1 × 2 multimode switch based on the MRRs^13^. The mode crosstalk (XT) ranging from −16.8 to −24.0 dB and the measured insertion loss (IL) of 5.4–9.1 dB can be achieved for four different channels. However, the MRRs may degenerate the operating bandwidth due to the critical resonating-condition. In addition, this approach is based on the relatively complicated demultiplexing-switching-multiplexing (DSM) process: the input multimode signals are demultiplexed to the fundamental modes and then switching them by using the single-mode (SM) switches; finally, these SM signals are multiplexed to the desired output modes. Yang *et al*. proposed a general architecture for on-chip mode switching and demonstrated a thermo-optic (TO) 2 × 2 four-mode switch based on the DSM process^22^. In order to simplify the MSS configuration, Sun *et al*. presented a 2 × 2 multimode switch, consisting of a pair of 1 × 1 MZI and TO based multimode switches and a pair of MRR based 2 × 2 SM switches^23^. A low IL of <1.2 dB and a low XT of <−16.6 dB can be measured for all channels and the footprint is 433 μm × 433 μm. Another approach could be the use of the MMI couplers to build an MSS. Priti *et al*. proposed and experimentally demonstrated an MSS based on the MMI couplers and TO phase-shifters^18^. A switching extinction ratio (ER) of >25 dB and a mode XT of <−12 dB were measured over the C-band. Xiong *et al*. demonstrated a 1 × 2 two-mode switch based on an MZI schematic and the electro-optic (EO) effect^24^. A short switching-time of <2.5 ns and the switching ER of 12.5–23.1 dB were experimentally achieved and the size of this mode switch is about 350 μm. Nevertheless, the traditional EO and TO effects in silicon, such as the Pockels effect and the Franz–Keldysh effect are relatively small, which would lead to a big size of the EO or TO phase-shifter to achieve the essential phase-transition^25^.

In order to increase the light-matter-interaction (LMI) inside the silicon switch, the TWC based configurations incorporated with phase-change materials (PCMs) and transparent conducting oxides (TCOs), including Ge_2_Sb_2_Te_5_ (GST)^26^, Ge_2_Sb_2_Se_4_Te_1_ (GSST)^19^, indium-tin-oxide (ITO)^20^, have been emerging as a promising approach to achieve an ultra-compact, broadband, and low-loss MSS. A nonvolatile and ultra-low-loss reconfigurable mode (De)MUX/switch has been reported based on a silicon TWC with the GSST-PCM^19^, which can achieve a compact coupling length of only 29.3 μm, a broad bandwidth covering S + C + L band, ultra-low ILs of 0.10 and 0.68 dB, and high mode ERs of 18.98 and 22.18 dB at “OFF” and “ON” states, respectively. Benefitting from the unique property of the epsilon-near-zero (ENZ) effect of the ITO-TCO, a reconfigurable mode (De)MUX/switch was numerically proposed by using a silicon TWC incorporated with a vertical metal-oxide-semiconductor (MOS) capacitor and an ITO layer^20^. A more compact length of 8.429 μm, an ultra-high switching-speed of 0.781 THz, and a low power-consumption of 11.74 fJ/bit can be achieved based on the carrier accumulation at the ENZ point. A silicon reconfigurable add/drop filter was demonstrated based on a vertical Si–SiO_2_–graphene capacitor^27^, which can overcome the need for continuous heating to keep switching state. The vertical MOS-capacitor is based on the vertical multilayers, which can be deposited in sequence and then etched together. The horizontal MOS-capacitor is based on the lateral multilayers, which requires the multi-depositing and multi-etching processes^28^. The advantages of the vertical MOS-capacitor are as follows: the vertical MOS-capacitor is easier to fabricate and can be operating for the quasi-TM mode as a horizontal slot is implemented. However, the vertical stacking structure suffers from the limited design-flexibility and may not be compatible with other on-chip components. In that sense, it would be preferred to design a carrier-accumulation based silicon MSS via a horizontal MOS-capacitor.

**Figure 1:**
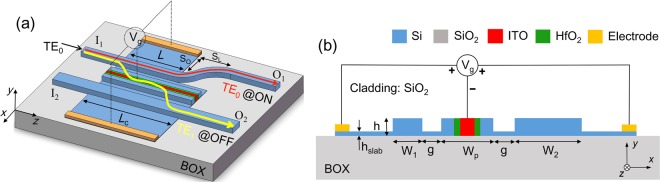
**(a)** Schematic illustration of the proposed silicon mode-selective switch based on a horizontal MOS-capacitor incorporated with epsilon-near-zero (ENZ) indium-tin-oxide (ITO). The capacitor is placed as a central waveguide of the triple-waveguide coupler. An S-bend waveguide is implemented to further reduce the mode crosstalk. **(b)** Cross-section of the Si/HfO_2_/ITO/HfO_2_/Si MOS-capacitor incorporated in the triple-waveguide coupler. The whole device is covered with the silica cladding and is based on the rib waveguide, but only P and P++ doped Si-slabs are shown in (**a**). The height of the slab of the silicon rib waveguide is chosen to be h_slab_ = 30 nm. The 1 × 10^18^ cm^−3^ doping concentration of P-doped Si is chosen for the triple-waveguide coupler to lower the electrical access resistance and optical propagation loss. The 1 × 10^20^ cm^−3^ doping concentration of P++ doped Si is chosen for the Si slabs positioned 1.0 μm away from the centre of two outer waveguides to achieve ohmic contacts with the electrodes.

In this paper, we propose and optimise a TWC based silicon MSS, consisting of two silicon outer waveguides (WGs) and a central horizontal MOS-capacitor incorporated with the ENZ-ITO, as shown in Fig. 1. A double-carrier-accumulation scheme offers the large phase-change and high switching-efficiency by using the horizontal Si/HfO_2_/ITO/HfO_2_/Si MOS-capacitor. This paper is organised as follows. In Section II-A, the schematic and principle of the proposed MSS are described in detail. In Section II-B, we study the electrical properties of the ITO material to reveal the carrier-accumulation based ENZ effect. In Section II-C, the modal characteristics of the MOS-type mode and the phase-matching condition are investigated for both “OFF” and “ON” states. In Section II-D, the operation and performance of MSS are presented and studied.

## Results

### Schematic and principle

The schematic diagram of the proposed MSS based on a silicon TWC is shown in Fig. 1(a), consisting of a central horizontal-MOS-capacitor incorporated with ENZ-ITO, an input WG with input port I_1_ and output port O_1_, and a bus WG with input/output ports I_2_/O_2_. The length of the MOS-capacitor is equal to the coupling length of the TWC, denoted by *L*_c_ in Fig. 1(a). In order to minimise the mode XT, an S-bend WG with the offset of S_o_ × S_L_ is implemented in between the straight input- and output-sections of the input WG. The length of the straight section of the input WG is denoted by *L*. The cross-section of the TWC is shown in Fig. 1(b), in which the widths of the input, central, and bus WGs are represented by W_1_, W_p_, and W_2_, respectively. The gap between the central and input/bus WGs is denoted by g. The whole device is covered with the SiO_2_ cladding and is based on the rib WG. The heights of the rib WG and the slab are chosen to be h = 220 nm and h_slab_ = 30 nm, respectively. To be clear, only the P and P++ doped silicon slabs are shown in Fig. 1(a). The heavily doped silicon would lead to an increased propagation loss. For the silicon WGs connected to the doped slabs, the lightly P-doping concentration is chosen to be 1 × 10^18^ cm^−3^ to lower the electrical access resistance and optical propagation loss. The measured propagation loss due to 1 × 10^18^ cm^−3^ doping was about 3.4 dB/mm^25^. Hence, an additional propagation-loss would be induced depending on both the doping and device length. A compact coupling length is preferred to reduce the propagation loss due to doping. For the slabs connected to the electrodes, the heavily P++ doping concentration of 1 × 10^20^ cm^−3^ is chosen to achieve ohmic contacts with the electrodes. In order to circumvent the influence of the electrodes and P++ doped slabs on the optical performance, they are placed 1.0 μm away from the centre of two outer WGs. As the whole device is covered with the SiO_2_ cladding, the ITO deposition window is opened using the second e-beam lithography process. An ITO film is then sputtered on the wafer and then the lift-off process is used to remove the ITO outside the deposition window. Finally, the electrical contact for ITO can be formed along with the waveguide. As the width of the ITO section was chosen as W_ITO_ = 50 nm as stated in Section II-C, the mode field in the upper ITO surrounded by cladding is cutoff, thereby the effect to the optical characteristics would be relatively slight. The non-ideal etched sidewalls would induce a strong optical absorption. The sidewalls of the vertical slot waveguides filled with ITO materials should be etched smoothly and accurately to avoid the optical absorption.

**Figure 2:**
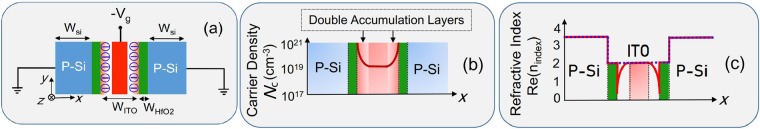
(**a**) Schematic of the Si/HfO_2_/ITO/HfO_2_/Si MOS-capacitor. When a negative gate-voltage is applied on the ITO layer, electrons accumulate at both the right and left ITO/HfO_2_ interfaces to form double accumulation-layers. (**b**) Carrier density distribution within the accumulation layers in ITO along *x* coordinate. (**c**) Real part of refractive-index distribution in Si, HfO_2_, and ITO along *x* coordinate. The dash blue and solid red lines are corresponding to the “OFF” and “ON” states, respectively.

The horizontal MOS-capacitor is comprised of the stacked Si/HfO_2_/ITO/HfO_2_/Si layers, as illustrated in Fig. 1(b). The refractive indices of the Si, SiO_2_, and HfO_2_ are set to be 3.47548, 1.46, and 1.98, respectively. The detailed configuration of the MOS-capacitor is shown in Fig. 2(a). The widths of the ITO, HfO_2_, and Si layers are denoted by W_ITO_, W_HfO2_, and W_si_, respectively. When a negative gate-voltage is applied on the ITO layer, electrons accumulate at both the right and left ITO/HfO_2_ interfaces to form double accumulation-layers (ACLs) and then the carrier density will be correspondingly increased inside two nanometer ACLs in the ITO, as shown in Fig. 2(b). Sequentially, the complex index of the ACLs in the ITO will be changed. It can be noted from Fig. 2(c) that the real part of the refractive index can be reduced to be close to near zero with an optimal applied gate-voltage at “ON” state, which can induce the ENZ effect. The operation principle of the proposed MSS is described as follows. (i) At “OFF” state, the phase-matching condition between three WGs of the TWC can be satisfied without any applied gate-voltage. The input quasi-TE_0_ mode can be multiplexed to the quasi-TE_1_ mode of the bus WG. (ii) At “ON” state with a negative gate-voltage on the ITO layer, the large index-change of the central MOS-type mode can be achieved due to the ENZ effect and then the phase-matching condition will be destructed. The input quasi-TE_0_ mode will be switched back into the input WG and outputs at port O_1_. Once the device is in the “ON” or “OFF” state, the capacitive effect does not need electrical current flow to keep the operating state. In addition, the proposed schematic could be exploited as a modulator instead of switch.

### Electrical characteristics of ENZ-ITO

The electrical characteristics of the ENZ-ITO are critical to the proposed double-carrier-accumulation based silicon MSS. The permittivity of the ITO layer can be calculated by using the well-known Drude-Lorentz model^20^:

1 $$\varepsilon ={\varepsilon }_{1}+j{\varepsilon }_{2}={\varepsilon }_{\infty }-\frac{{\omega }_{p}^{2}}{\omega (\omega +j{\rm{\gamma }})}$$

2 $${\omega }_{p}^{2}=\frac{{N}_{c}{e}^{2}}{{\varepsilon }_{0}{m}^{\ast }}$$

where *ε*_∞_ = 3.9 is the permittivity of the high-frequency ITO; *ω* is the angular momentum in rad/s; γ = 1.84 × 10^14^ rad/s is the electron scattering rate; *ω*_*p*_ is the plasma frequency; *ε*_0_ is the vacuum permittivity; *e* is the elementary electron charge; m^*^ = 0.35 *m*_0_ is the effective mass of the electron; *m*_0_ is the rest mass of the electron; *N*_*c*_ is the carrier concentration.

**Figure 3:**
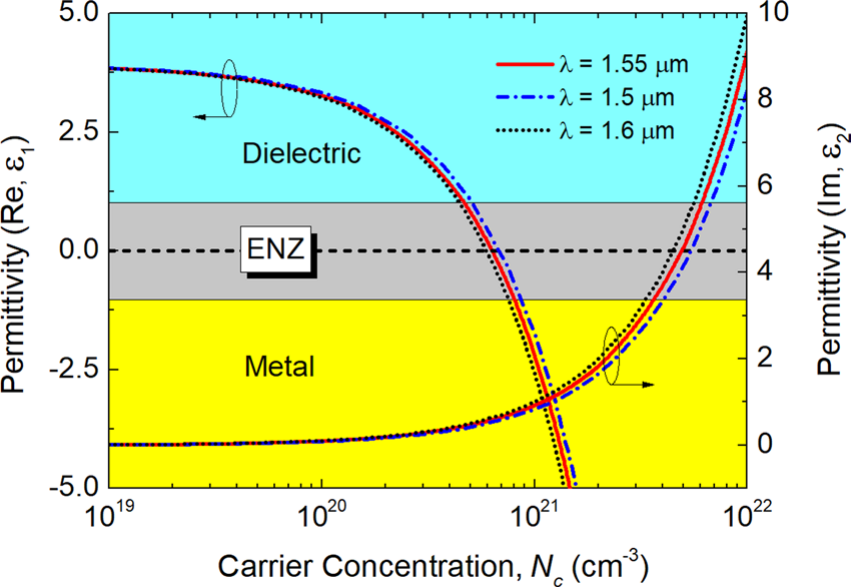
Variations of the real part (left *y*-axis) and imaginary part (right *y*-axis) of the complex permittivity of ITO with different carrier concentrations at the wavelengths of λ = 1500, 1550, and 1600 nm, respectively based on the Drude-Lorentz model. The ENZ region is highlighted by the gray area under the ITO real permittivity in between −1.0 and 1.0. With the increase of the carrier concentration, the state of the ITO can gradually turn from the “dielectric” state to the “quasi-metallic” state and finally to the metallic state.

Variations of the real part (left *y*-axis) and imaginary part (right *y*-axis) of the complex permittivity of the ITO with the carrier concentration are shown in Fig. 3 at the wavelengths of 1500 nm (dash-dotted blue line), 1550 nm (solid red line), and 1600 nm (dotted black line), respectively. It can be noted that the real permittivity is decreased with the increase of the carrier concentration, while the imaginary permittivity is increased. Accordingly, the ITO state would be changed from the dielectric state to the “quasi-metallic” state and finally to the metallic state, which are denoted by the light-blue, gray, and yellow regions in Fig. 3. The ENZ region is under the ITO real-permittivity in between −1.0 and 1.0.

Next, both the carrier concentration and the thickness of the ACL are investigated with respect to the applied gate-voltage. The carrier concentration in the ITO-ACL can be estimated by the following formula^29^:

3 $${N}_{c}={N}_{d}+\frac{{\varepsilon }_{{\rm{0}}}\cdot {\varepsilon }_{{{\rm{HfO}}}_{2}}\cdot {{\rm{V}}}_{g}}{e\cdot {{\rm{W}}}_{{{\rm{HfO}}}_{2}}\cdot {{\rm{W}}}_{acc}}$$

where *ε*_HfO2_ = 25 is the DC permittivity of the HfO_2_; V_g_ is the applied gate-voltage; *N*_d_ is the intrinsic carrier density of the bulk ITO film. In this case, an electron carrier-concentration of *N*_*d*_ = 10^19^ cm^−3^ is chosen for the ITO layer to match its real permittivity with that of its adjacent HfO_2_ layer. For the width of the HfO_2_ layer, W_HfO2_, it can be noted from Eq. (3) that a thinner HfO_2_ layer would induce a lower gate-voltage. However, the MOS capacitance would also be increased with a thinner HfO_2_ layer, which would reduce the switching speed. Hence, the width of the HfO_2_ layer should be chosen to balance the power consumption and the switching speed. In this work, the width of the HfO_2_ layer is chosen to be W_HfO2_ = 10 nm. W_acc_ is the width of the ACL. There are several papers^30^ assuming that the ACL is equivalent to be with the uniform concentration and with the certain ACL-thickness ranging from 1.0 to 10 nm. A thicker ACL would provide a higher switching-efficiency benefiting from the enhancement of the LMI inside the device. Actually, the accurate carrier distribution should be experimentally measured. In this work, different ACL thicknesses, W_acc_, will be studied to reveal their effects on the switching performance.

**Figure 4:**
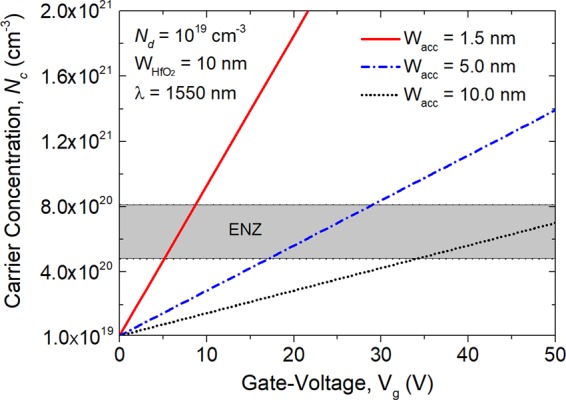
Variations of the carrier concentration in the ACLs with the applied voltage and ACL thickness, W_acc_ = 1.5, 5.0, and 10.0 nm, respectively at the wavelength of 1550 nm. The ENZ region is highlighted by the gray area under the ITO real-permittivity in between −1.0 and 1.0, corresponding to the carrier concentration in between *N*_c_ = 4.8 × 10^20^ and 8.135 × 10^20^ cm^−3^.

Variations of the carrier concentration in the ACLs with the applied voltage at the wavelength of 1550 nm are calculated for the ACL thickness, W_acc_ = 1.5, 5.0, and 10.0 nm, respectively, as shown in Fig. 4. The ENZ region is with the ITO real-permittivity in between −1.0 and 1.0, which is also corresponding the carrier concentration in between *N*_c_ = 4.8 × 10^20^ and 8.135 × 10^20^ cm^−3^. It can be noted from Fig. 4 that the applied gate-voltage for the ENZ region is decreased with the decrease of the width of the ACL. Hence, the power consumption and the switching-efficiency should be balanced according to the ACL width.

### MOS-Type mode and phase-matching

**Figure 5:**
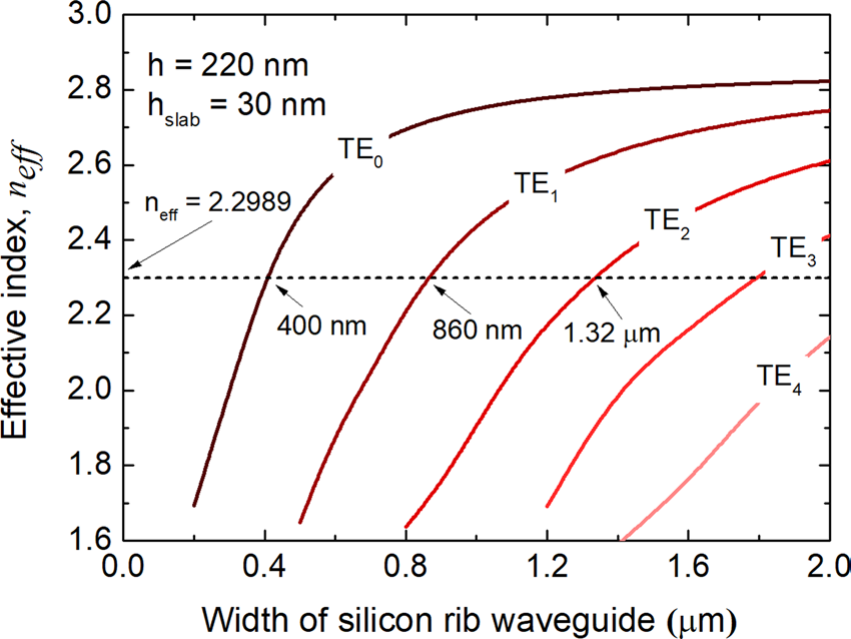
Variations of the effective index with the width of the silicon rib waveguide. Here, the heights of the slab and the silicon rib waveguide are set as h_slab_ = 30 nm and h = 220 nm, respectively. The effective index of the input waveguide with size of 220 nm × 400 nm is calculated to be 2.2989 using FV-FEM, denoted by a horizontal dashed-black line.

The MOS-type mode of the proposed horizontal MOS-capacitor plays a key role in the silicon MSS. In this Section, the isolated modes of the silicon WGs, supermodes of the TWC, and MOS-type mode of the central MOS-capacitor are studied by using the full-vectorial finite element method (FV-FEM). The coupling length, *L*_c_, is optimised according to the phase-matching condition at “OFF” state. The effective indices of the isolated silicon rib-WGs are calculated and shown in Fig. 5. The effective index of the quasi-TE_0_ mode of the input rib-WG with the size of h × W_1_ = 220 nm × 400 nm is calculated to be *n*_*eff*_ = 2.2989, denoted by a horizontal dashed-black line in Fig. 5. The phase-matched widths are chosen to be 860 nm and 1.32 μm for the quasi-TE_1_ and quasi-TE_2_ modes, respectively. In this case, the MSS for handling the quasi-TE_0_ and quasi-TE_1_ modes is considered as an example to explain the operation principle. But, even higher-order modes can also be handled based on the proposed mechanism.

**Figure 6:**
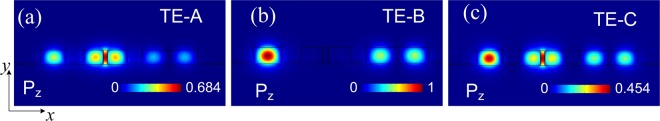
Supermode fields of the phase-matched triple-waveguide coupler based on the FV-FEM simulation. Poynting vector, P_z_ (*x*, *y*) field profiles of (**a**) TE-A, (**b**) TE-B, and (**c**) TE-C supermodes. Here, parameters for FV-FEM simulation: W_1_ = 400 nm, W_2_ = 860 nm, W_ITO_ = 50 nm, W_HfO2_ = 10 nm, W_si_ = 307.5 nm, h = 220 nm, h_slab_ = 30 nm, and g = 200 nm.

**Figure 7:**
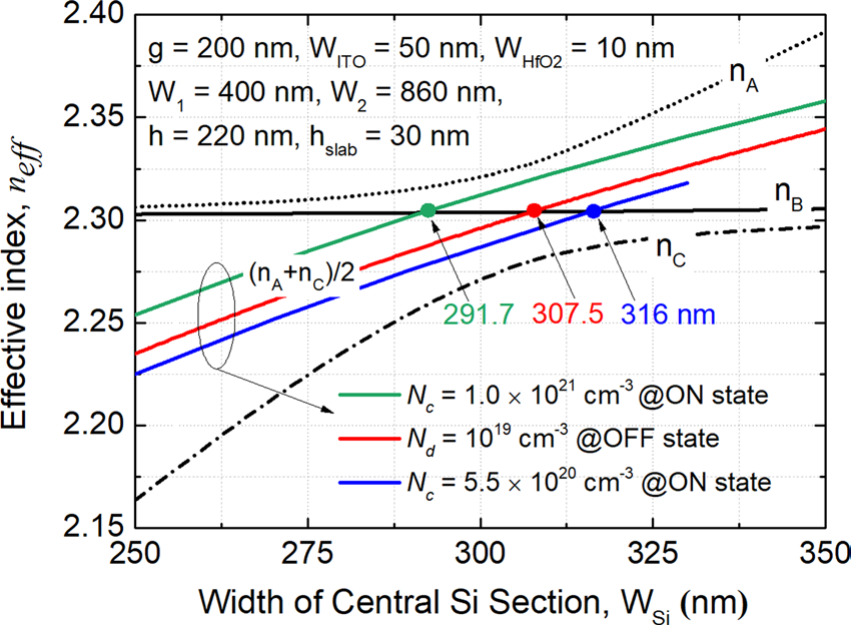
Variations of the effective indices of the supermodes with the width of the central silicon-section, W_si_ based on the FV-FEM simulation. The solid green, red, and blue lines are corresponding to the carrier concentrations of *N*_c_ = 1 × 10^21^, 1 × 10^19^, and 5.5 × 10^20^ cm^−3^ for two accumulation layers (W_acc_ = 1.5 nm), respectively.

Although the phase-matched widths of the input and bus WGs can be obtained based on the isolated mode-condition, that of the MOS-capacitor based central WG needs to be investigated based on the supermodes and phase-matching condition of the TWC at “OFF” state. The supermodes of the TWC are calculated by using the FV-FEM and the Poynting vector, P_z_ (*x*, *y*) field profiles of three supermodes for handling the quasi-TE_0_ and quasi-TE_1_ modes are shown in Fig. 6, denoted by the TE-A, TE-B, and TE-C, respectively. As stated earlier, the phase-matching condition should be satisfied at “OFF” state, while that at “ON” state should be destructed as much as possible. To meet the phase-matching condition of the TWC, the effective indices of three supermodes must satisfy the formula^20^: n_A_ + n_C_ = 2n_B_, where n_A_, n_B_, and n_C_ are the effective indices of the TE-A, TE-B, and TE-C supermodes, respectively. Variations of the supermodes effective-indices with the width of the central silicon-section, W_si_ are shown in Fig. 7. It can be noted that the effective index of the TE-B supermode almost keeps constant with the change of the W_si_, while those of the TE-A and TE-C supermodes are increased. It can be observed from Fig. 6 that the TE-B mode-field is only confined in the input and bus WGs, which cannot be influenced by the variation of the central WG. However, there are major mode-fields of both the TE-A and TE-C supermodes confined in the MOS-capacitor based central WG. Hence, the phase-condition of the TWC can be adjusted according to different carrier-concentrations inside the MOS-capacitor. It can also be noted from Fig. 7 that the phase-matched width can be changed from W_si_ = 307.5 nm at “OFF” state to 316 and 291.7 nm for *N*_c_ = 1 × 10^21^ and 5.5 × 10^20^ cm^−3^ at “ON” state, respectively. In next Section, the carrier concentration will be optimised according to the performance of the optimal design at “ON” state.

**Figure 8:**
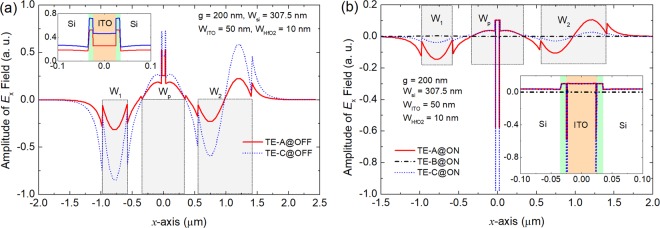
1D plot of the amplitude of *E*_*x*_ fields of the supermodes at **(a)** “OFF” and **(b)** “ON” states, respectively at λ = 1550 nm. The insets show the enlarged views of the *E*_*x*_ field profiles in the ITO/HfO_2_ regions generated by the FV-FEM. The electric field density is mainly confined in the HfO_2_ layers at “OFF” state and shifts to the strongly absorbing accumulation-layers at “ON” state with *N*_c_ = 5.5 × 10^20^ cm^−3^, which can lead to the large refractive-index modulation.

In order to further explain the mechanism of the MOS-type mode based MSS, 1D plots of the amplitude of the *E*_*x*_ fields of the supermodes are illustrated in Fig. 8(a,b) for the “OFF” and “ON” states, respectively. As the MOS-type modes are only existed in the TE-A and TE-C supermodes, shown in Fig. 6, the *E*_*x*_ fields of these two supermodes are presented in Fig. 8(a). At “OFF” state, the *E*_*x*_ fields of both supermodes in the MOS-capacitor are mainly confined in two HfO_2_ layers, as shown in the inset of Fig. 8(a). This can be explained that the *E*_*x*_ field undergoes a large discontinuity due to the big index-difference between the silicon and oxide layers, which can make the field mainly confined in the low-index oxide slot. The oxide slot is comprised of the HfO_2_/ITO/HfO_2_ layers. Although an optimal electron carrier-concentration is chosen to match the real index between the HfO_2_ (1.98) and ITO (1.96 + 0.003*i*) at “OFF” state, the *E*_*x*_ field still undergoes the discontinuity due to the different imaginary-indices and is mainly confined in the HfO_2_ layers. It can be noted from Fig. 8(b) that the *E*_*x*_ fields of the TE-A and TE-C supermodes are squeezed into the ACLs at “ON” state. The enlarged view of the *E*_*x*_ fields in the oxide layers is shown in Fig. 8(b) as an inset. As the ACLs turn to the “quasi-metallic” state at “ON” state, the “oxide slot” is comprised of the HfO_2_/ACL/ITO/ACL/HfO_2_ layers, which can squeeze the *E*_*x*_ fields into the ultra-low-index ACLs. Therefore, the phase-condition can be significantly changed at “ON” state.

**Figure 9:**
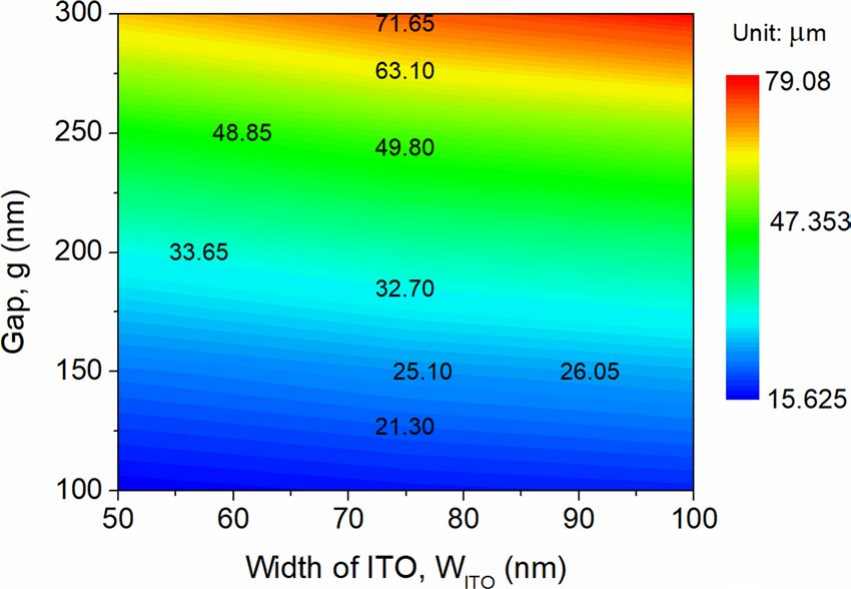
Variations of the coupling length with both the gap and width of the ITO under the phase-matching condition based on the FV-FEM. Here, parameters for FV-FEM simulation: W_HfO2_ = 10 nm, W_1_ = 400 nm, W_2_ = 860 nm, h = 220 nm, and h_slab_ = 30 nm. The phase-matched width of the central silicon-section, W_si_, should be adjusted accordingly to meet the phase-matching condition.

In this case, the device length is depended on the coupling length, *L*_c_, which can be calculated based on the formula^20^: *L*_c_ = λ/2(n_A_ − n_B_). Variations of the coupling length with both the gap and width of the ITO are shown in Fig. 9 under the phase-matching conditions. It can be noted from Fig. 9 that the coupling length is increased with the increases of both gap and W_ITO_. As a compact *L*_c_ is preferred, a smaller gap and a narrower width of the ITO layer would be better. The width of the ITO layer is W_ITO_ = 50 nm considered in this case.

### Operation and performance of mode-selective switch

In this Section, the propagation performance of the proposed MSS is studied by using the three-dimensional full-vectorial eigenmode expansion (3D-FV-EME) method. In this case, the mesh size of the MOS-capacitor is extremely critical for the rigorous simulation due to the thickness of the ACLs in between 1.0 nm to 10 nm. For the HfO_2_/ITO/HfO_2_ layers, an extremely small mesh-size of 0.1 nm was applied in the *x*-axis and 10 nm was used for the y-axis. For the other regions in the computation window, the mesh sizes in both the *x*- and y-axes were set to be 10 nm. An MSS with the gap of g = 200 nm is studied firstly and then variations of both the gap and ACL thickness are also investigated in detail.

**Figure 10:**
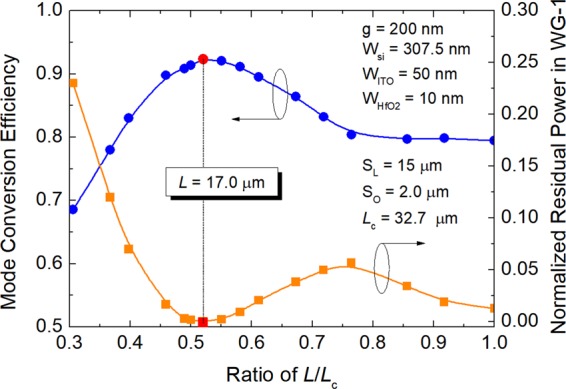
Variations of the mode conversion efficiency (left *y*-axis) and normalised residual power in the input waveguide (right *y*-axis) at “OFF” state with the ratio between the length of the straight input-waveguide-section and the coupling length, *L*/*L*_c_. The optimal results with *L* = 17.0 μm are denoted by red dots, which are calculated by using the 3D-FV-EME method.

In order to minimise the mode XT, an S-bend WG with the size of S_o_ × S_L_ = 2.0 μm × 15 μm is implemented in the input WG, as shown in Fig. 1(a). The position of the S-bend WG is optimised according to the mode XT by using the 3D-FV-EME method. Variations of the mode conversion efficiency (left *y*-axis) and normalised residual power in the input WG (right *y*-axis) at “OFF” state with the ratio between the length of the straight input-waveguide-section and the coupling length, *L*/*L*_c_, are shown in Fig. 10 for g = 200 nm and phase-matched W_si_ = 307.5 nm. It can be noted that the optimal length of the straight section of the input WG is chosen to be *L* = 17.0 μm for the ratio, *L*/*L*_c_ = 0.52 close to the midpoint of the input WG. The mode conversion efficiency and the normalised residual-power in the input WG can achieve to be 0.9238 and 1.725 × 10^−4^, respectively. The mode XT, calculated based on XT = −(ER − IL)^21^, can be dramatically decreased to be −37.63 dB by introducing an S-bend WG. The ER and IL are calculated to be 37.29 and −0.34 dB, respectively at “OFF” state.

**Figure 11:**
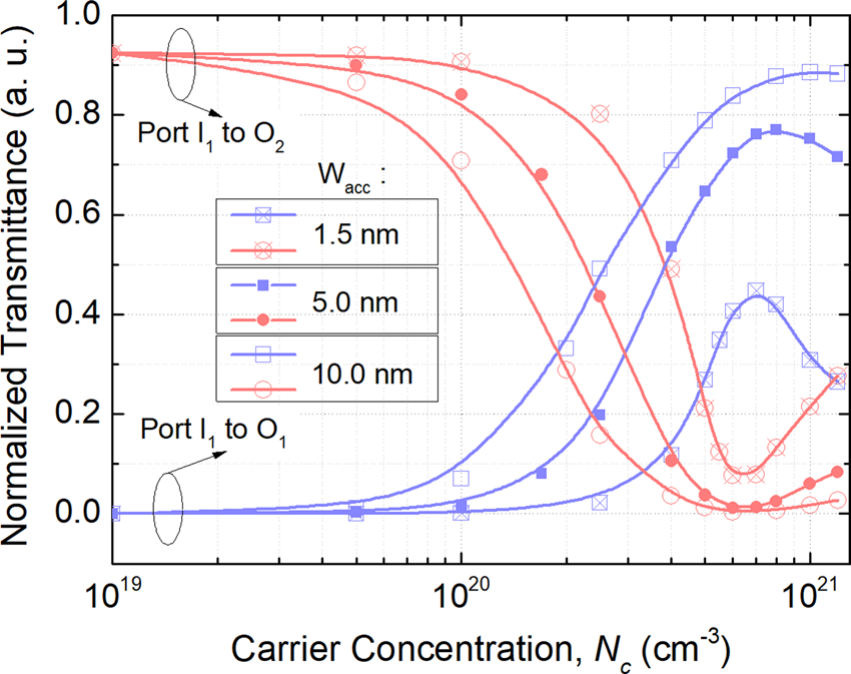
Variations of the normalised transmittance at “ON” state with the carrier concentration for gap, g = 200 nm and ACL thickness, W_acc_ = 1.5, 5.0, and 10.0 nm, respectively. The 3D-FV-EME method is used for the simulation.

**Figure 12:**
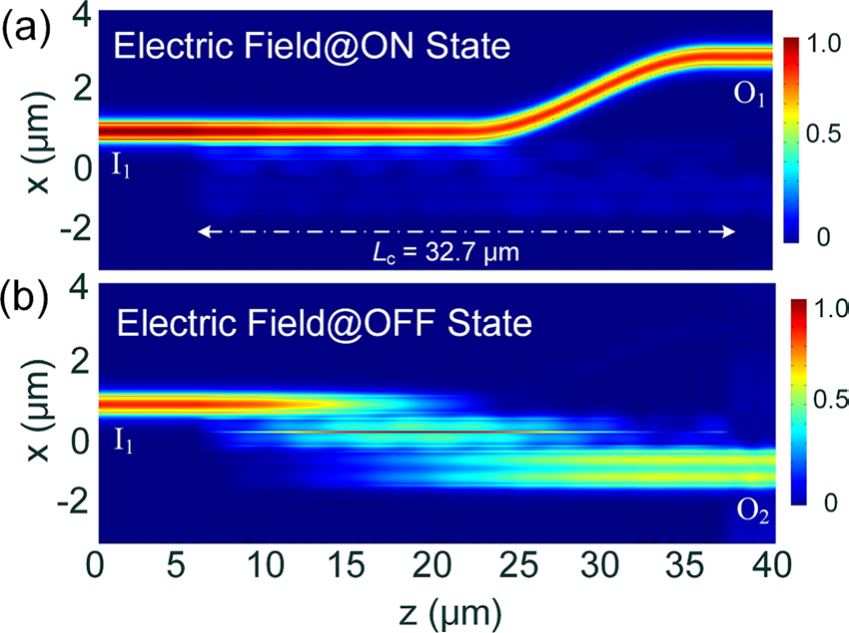
Propagation electric fields, |E|, along z-coordinate at **(a)** “ON” and **(b)** “OFF” states, respectively. Here, the parameters for 3D-FV-EME simulation: g = 200 nm, W_1_ = 400 nm, W_2_ = 860 nm, W_ITO_ = 50 nm, W_HfO2_ = 10 nm, W_si_ = 307.5 nm, h = 220 nm, h_slab_ = 30 nm, and *L*_c_ = 32.7 μm.

**Figure 13:**
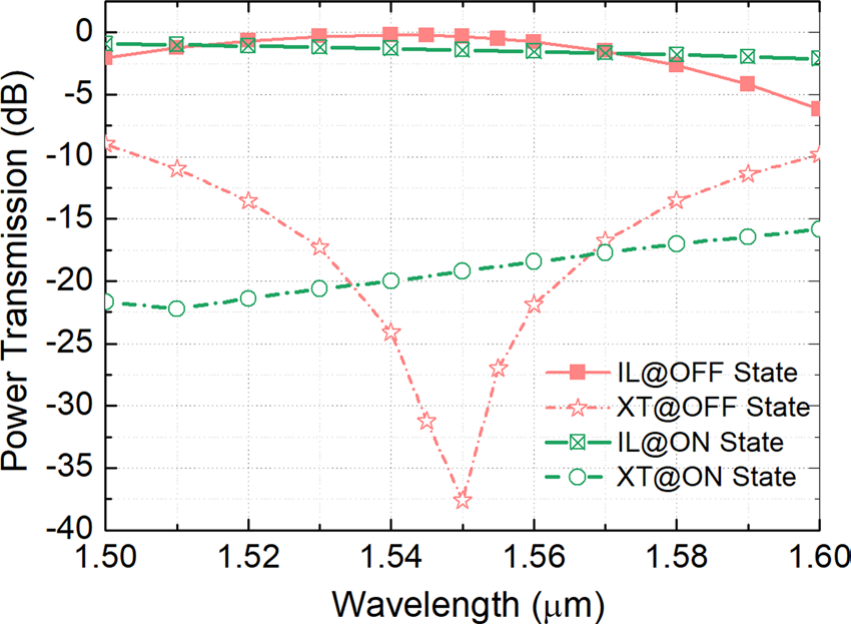
Wavelength dependence of the optimised mode-selective switch for both “OFF” and “ON” states based on the 3D-FV-EME method.

Next, the effects of the ACL thickness, W_acc_, and the gap, g, on the switching performance are investigated at “ON” state. Variations of the normalised transmittance with the carrier concentration are calculated by using the 3D-FV-EME method and shown in Fig. 11 for the gap, g = 200 nm and the ACL thickness, W_acc_ = 1.5, 5.0, and 10.0 nm, respectively at “ON” state. The power transmitted from the port I_1_ to ports O_1_ and O_2_ are denoted by the red and blue lines, respectively. It can be noted that with the increase of the carrier concentration, the power from port I_1_ to O_2_ is decreased and then increased after the optimal *N*_c_, while that to port O_1_ is increased and then decreased. At the optimal *N*_c_ = 6.0 × 10^20^ cm^−3^, the lowest mode XTs are −11.13, −19.21, and −24.42 dB for W_acc_ = 1.5, 5.0, and 10.0 nm, respectively. Hence, the switching performance is increased with the increase of the ACL thickness, which should be obtained from the experimental results. As stated in reference^31^, the ACL thickness was experimentally measured to be 5 ± 1 nm for all applied voltages, which has been cited and used for many experimental and theoretical cases. For W_acc_ = 5.0 nm and g = 200 nm, the mode XT, ER, and IL are calculated to be −19.21, 17.8, and −1.4 dB at “ON” state. The propagation electric fields, |E|, along z-axis at “ON” state is shown in Fig. 12(a), which shows the input quasi-TE_0_ mode is propagating along the input WG and outputs at port O_1_. Although the IL is −1.4 dB at “ON” state, the power coupled to the bus WG is as small as 1.2%. The reason is that the extra power is absorbed by the “quasi-metallic” ACL layers. For the “OFF” state, the propagation field is also calculated and shown in Fig. 12(b). It can be noted that the input quasi-TE_0_ mode is completely multiplexed to the quasi-TE_1_ mode of the bus WG. The wavelength dependence of the optimised MSS is calculated by using the 3D-FV-EME method and shown in Fig. 13 for both “OFF” and “ON” states. It can be noted that 3 dB bandwidths are 82.5 and 100 nm for the “OFF” and “ON” states, respectively. With the mode XT of <−15.0 dB, the bandwidth at “OFF” state is calculated to be over 52.3 nm from 1523.5 to 1575.8 nm. At “ON” state, the mode XT is lower than −15.85 dB over a 100 nm bandwidth.

**Figure 14:**
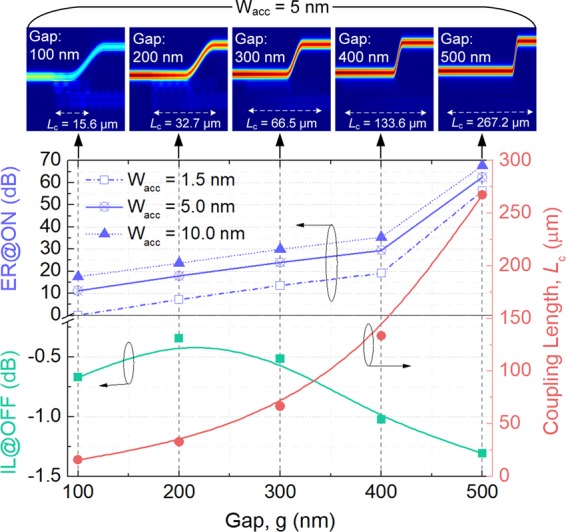
Variations of the extinction ratio at “ON” state (upper-half of left *y*-axis), insertion loss at “OFF” state (lower-half of left *y*-axis), and the coupling length, *L*_c_ (right *y*-axis) with the gap, g for W_acc_ = 1.5, 5.0, and 10.0 nm, respectively^30^. Five panels on the top are corresponding to the propagation electric fields, |E|, along z-coordinate at “ON” state for W_acc_ = 5 nm and g = 100, 200, 300, 400, and 500 nm, respectively. The 3D-FV-EME method was used for the simulation.

Although the performance of the optimised MSS with the ACL-thickness, W_acc_ = 5.0 nm and gap, g = 200 nm has been studied, variations of the device performance are also investigated with respect to both the ACL-thickness and gap. Variations of the ER at “ON” state (upper-half of left *y*-axis), IL at “OFF” state (lower-half of left *y*-axis), and the coupling length, *L*_c_ (right *y*-axis) with the gap are calculated based on the 3D-FV-EME method for W_acc_ = 1.5, 5.0, and 10.0 nm, respectively^30^. In the simulation, the carrier concentration was set to be *N*_c_ = 6.0 × 10^20^ cm^−3^ and the corresponding gate-voltages are 6.4, 21.35, and 42.7 V for W_acc_ = 1.5, 5.0, and 10.0 nm, respectively. The parameters of the phase-matched W_si_, *L*_c_, and *L* were optimised according to the phase-matching conditions. It can be noted from Fig. 14 that the mode ER is increased with the increase of the gap. The mode ER for W_acc_ = 5 nm is increased from 11.13 to 62.48 dB with the gap changing from g = 100 to 500 nm. The corresponding propagation electric-fields, |E|, are shown as five panels in Fig. 14. However, as the gap is increasing, the coupling length is also going to be longer. The coupling lengths are calculated to be 15.6, 32.7, 66.5, 133.6, and 267.2 μm for g = 100, 200, 300, 400, and 500 nm, respectively. Another issue is the IL at “OFF” state, which would also be enhanced with the increase of the gap (>200 nm), as shown in the lower-half of left *y*-axis of Fig. 14. The reason can be explained that with the increased coupling-length for the larger gap, the absorption loss would be increased due to the imaginary index (0.003) of the ITO at “OFF” state. However, for the gap, g = 100 nm, the IL is also slightly increased due to the incomplete coupling-power in the input WG. Therefore, we can state that a large-enough mode ER at “ON” state can be achieved with a wide gap for any ACL-thickness, with the sacrifices of the coupling length and the IL at “OFF” state. It can be noted from Fig. 14 that for W_acc_ = 1.5 nm, the mode ERs at ON state are 7.22, 13.56, 19.05, and 56.36 dB for gap, g = 200, 300, 400, and 500 nm, respectively. Meanwhile, the IL at “OFF” and “NO” states are −0.34 dB and −3.9 dB, −0.51 dB and −2.1 dB, −1.0 dB and −1.19 dB, −1.3 dB and −0.63 dB for g = 200, 300, 400, and 500 nm, respectively. For W_acc_ = 5.0 nm, the mode ERs and IL at “ON” state are 17.8 dB and −1.41 dB, 23.9 dB and −0.71 dB, 29.46 dB and −0.38 dB, 62.48 dB and −0.19 dB for g = 200, 300, 400, and 500 nm, respectively. For W_acc_ = 10.0 nm, the mode ERs and IL at “ON” state are 23.7 dB and −0.76 dB, 29.89 dB and −0.38 dB, 35.4 dB and −0.19 dB, 67.72 dB and −0.1 dB for g = 200, 300, 400, and 500 nm, respectively.

The mode switching speed is limited to the RC delay. The MOS capacitance is calculated based on the formula^20^:

4 $$C=\frac{{\varepsilon }_{0}\cdot {\varepsilon }_{{{\rm{HfO}}}_{2}}\cdot h\cdot {L}_{C}}{{{\rm{W}}}_{{{\rm{HfO}}}_{2}}}$$

The power-consumptions per bit can be calculated from^32^: *E/bit* = CV_g_^2^*/*2. The resistivity of the HfO_2_, doped silicon and ITO are set to be 10^12^, 2.36 and 5 × 10^−4^ Ω•m according to the reported references^33–35^. The internal impedances are calculated to be 244.5, 1008.8, and 1008.8 Ω for W_acc_ = 1.5, 5.0, and 10.0 nm, respectively based on the equivalent RC circuit of our proposed MOS-capacitor. Considering the resistivity and MOS-capacitor structure, the operation bandwidth (*BW*) can be obtained by *BW* = 1*/RC*. The optical and electro-optic performance are summarised in Table 1. For the ACL width, W_acc_ = 5 and 10 nm, the gap is chosen to be g = 200 nm for the high optical performance, whereas the gap is increased to be g = 400 nm for W_acc_ = 1.5 nm. The power-consumptions per bit are increased from tens of fJ/bit for W_acc_ = 1.5 nm to hundreds of fJ/bit for W_acc_ = 5.0 and 10.0 nm due to the larger gate-voltage for the wider ACL-width. The operation speeds are calculated to be 6.3, 6.2, and 6.2 GHz for W_acc_ = 1.5, 5.0, and 10.0 nm, respectively. Furthermore, the switching speed of our proposed device can be increased via reducing the internal impedance or the MOS capacitance.

**Table 1:** Optical and electro-optic performance of the optimised MSS.

W_acc_ (nm)	Gap (nm)	*L*_c_ (μm)	IL (dB)		ER (dB)		V_g_ (V)	*BW* (GHz)	*E/bit* (fJ/bit)
			@OFF	@ON	@OFF	@ON			
1.5	400	133.6	−1.02	−1.19	28.52	19.05	6.4	6.3	13.32
5	200	32.7	−0.34	−1.41	37.29	17.8	21.35	6.2	36.29
10	200	32.7	−0.34	−0.76	37.29	23.7	42.7	6.2	145.2

The requirement of the higher gate-voltage leads to a great challenge to the HfO_2_ layers, which would exceed the breakdown strength and raises the concern of reliability. The performance of our proposed device is also studied with respect to a low V_g_ of 2.5 V to prevent the HfO_2_ breakdown (5 MV/cm)^36^. As the applied V_g_ was set as 2.5 V, the carrier concentrations of the accumulation layers are calculated to be 2.4 × 10^20^, 7.9 × 10^19^, and 4.45 × 10^19^ cm^−3^ for three ACL thicknesses, W_acc_ = 1.5, 5.0, and 10.0 nm, respectively. We can note from Fig. 14 that a large-enough mode ER at “ON” state can be achieved with a wide gap, but the coupling length and the IL at “OFF” state would be increased. In order to achieve a reasonably high mode ER, a wide gap of g = 600 nm is chosen as an example for this 2.5 V Vg. The mode ERs at “ON” state are calculated to be 10, 7.7, and 7.2 dB for W_acc_ = 1.5, 5.0, and 10.0 nm, respectively. The coupling length and the IL at “OFF” state are increased to be *L*_c_ = 455 μm and IL = 1.87 dB, respectively. Moreover, the mode ER at “OFF” state is also deteriorated to be 13.0 dB. In addition, the mode ER at both “ON” and “OFF” states can be further increased via a wider gap between three waveguides, but a longer device would be induced and the loss would also be deteriorated. Hence, a high static permittivity for low power operation and a wide oxide-layer and gap would be preferred to prevent the breakdown and retain a high performance.

## Conclusion

In conclusion, a silicon-MSS has been proposed and optimised based on a horizontal MOS-capacitor incorporated with the ENZ-ITO. The double-carrier-accumulation effect was introduced to enhance the switching efficiency. The permittivity of the ITO as a function of the carrier concentration and the gate voltage has been calculated based on the Drude-Lorentz model. The modal characteristics of the MOS-type mode and supermodes of the TWC have been studied by using the FV-FEM for the phase-matching condition. The propagation performance of the proposed MSS was investigated by using the 3D-FV-EME method. The influence of the ACL-thickness and gap on the device performance has been analyzed. The optimised MSSes for switching the quasi-TE_0_ and quasi-TE_1_ modes have been achieved for W_acc_ = 1.5, 5.0, and 10.0 nm, respectively. With the increase of the gap, any desired mode-ER can be obtained regardless of the ACL thickness. The operation speed and power consumption can reach to 6.3 GHz and 13.32 fJ/bit, 6.2 GHz and 36.29 fJ/bit, 6.2 GHz and 145.2 fJ/bit for W_acc_ = 1.5, 5.0, and 10.0 nm, respectively. The insertion losses at “OFF” and “ON” states were −1.02 and −1.19 dB, −0.34 and −1.41 dB, −0.34 and −0.76 dB for these three ACL thicknesses, respectively. The mode ER at “OFF” and “ON” states have achieved to be 28.52 and 19.05 dB, 37.29 and 17.8 dB, 37.29 and 23.7 dB, respectively. The applied gate-voltage of 2.5 V under the HfO_2_ breakdown was also investigated. The proposed MSS can be used for efficient mode-routing to build the scalable MDM networks.

## Methods

The electrical characteristics of the epsilon-near-zero indium-tin-oxide (ENZ-ITO) are calculated based on the Drude-Lorentz model. The intrinsic carrier-concentration of *N*_*d*_ = 10^19^ cm^−3^ is chosen for the ITO bulk film. The metal-oxide-semiconductor (MOS)-type mode of the proposed horizontal MOS-capacitor is calculated by using the full-vectorial finite element method (FV-FEM). The mode characteristics of the isolated modes of the silicon WGs and supermodes of the triple-waveguide coupler (TWC) are also calculated based on the FV-FEM. The phase-matching condition of the TWC at “OFF” state is determined by using the FV-FEM. The coupling length is calculated based on the the formula^20^: *L*_c_ = λ/2(n_A_ − n_B_); where n_A_ and n_B_ are the effective indices of the TM-A and TM-B supermodes. The propagation fields, mode crosstalk, mode extinction-ratio, optical bandwidths, and insertion losses of the optimal mode selective switches (MSSes) are obtained by using the three-dimensional full-vectorial eigenmode expansion (3D-FV-EME) method. The operation bandwidth (*BW*) is calculated by *BW* = 1*/RC*. The power-consumptions per bit is calculated from^32^: *E/bit* = CV_g_^2^*/*2; where C is the MOS capacitance and V_g_ is the applied gate-voltage.
